# Identification of a Major QTL and Validation of Related Genes for Tiller Angle in Rice Based on QTL Analysis

**DOI:** 10.3390/ijms23095192

**Published:** 2022-05-06

**Authors:** Dan-Dan Zhao, Yoon-Hee Jang, Muhammad Farooq, Jae-Ryoung Park, Eun-Gyeong Kim, Xiao-Xuan Du, Rahmatullah Jan, Kyung-Hwan Kim, Soo In Lee, Gang-Seob Lee, Kyung-Min Kim

**Affiliations:** 1 Department of Applied Biosciences, Graduate School, Kyungpook National University, Daegu 41566, Korea; qx288mm@naver.com (D.-D.Z.); uniunnie@naver.com (Y.-H.J.); mfarooqsr@gmail.com (M.F.); dkqkxk632@naver.com (E.-G.K.); rehmatbot@yahoo.com (R.J.); 2 Crop Breeding Division, National Institute of Crop Science, Rural Development Administration, Wanju 55365, Korea; icd92@naver.com; 3 Department of Agricultural Biotechnology, National Institute of Agricultural Sciences, Rural Development Administration, Jeonju 54874, Korea; haobingshuaike@hotmail.com (X.-X.D.); biopiakim@korea.kr (K.-H.K.); silee@korea.kr (S.I.L.); 4 Coastal Agriculture Research Institute, Kyungpook National University, Daegu 41566, Korea

**Keywords:** plant architecture, rice, tiller angle, quantitative trait loci, auxin, breeding

## Abstract

An ideal plant architecture is an important condition to achieve high crop yields. The tiller angle is an important and complex polygenic trait of rice (*Oryza sativa* L.) plant architecture. Therefore, the discovery and identification of tiller angle-related genes can aid in the improvement of crop architecture and yield. In the present study, 222 SSR markers were used to establish a high-density genetic map of rice doubled haploid population, and a total of 8 quantitative trait loci (QTLs) were detected based on the phenotypic data of the tiller angle and tiller crown width over 2 years. Among them, four QTLs (*qTA9*, *qCW9*, *qTA9-1*, and *qCW9-1*) were overlapped at marker interval RM6235–RM24288 on chromosome 9 with a large effect value regarded as a stable major QTL. The selected promising related genes were further identified by relative gene expression analysis, which gives us a basis for the future cloning of these genes. Finally, *OsSAURq9*, which belongs to the *SMALL AUXIN UP RNA* (*SAUR*), an auxin-responsive protein family, was selected as a target gene. Overall, this work will help broaden our knowledge of the genetic control of tiller angle and tiller crown width, and this study provides both a good theoretical basis and a new genetic resource for the breeding of ideal-type rice.

## 1. Introduction

The rice plant type is determined by the tiller angle, leaf angle, tiller number, panicle architecture, and plant height in a multi-tiller crop [1]. Among them, the tiller angle determines the growth density and contributes to the grain yield of rice plants [2]. One of the key qualities for achieving the ideal plant type and increasing the yields of rice inbreeding is the tiller angle, which is the angle between the main stem and its side tillers [3]. Plants with relatively erect and vertical tillers are called compact type and plants with wide tillers are called spreading type [4]. The spreading type takes up too much space, causing shade and lodging and lowering the photosynthetic efficiency and grain yield [5]. By contrast, the compact type has a more effective plant architecture, given that such a small tiller angle leads to higher prospective yields [6]. Therefore, it is particularly important to identify more genetic loci and regulatory mechanisms related to the tiller angle, which can provide new approaches to increasing rice yields.

The tiller angle is controlled by QTLs, and the genetic loci associated with the tiller angle have been studied by QTL mapping in recent years [3,7,8]. However, cloned genes related to controlling the tiller angle are still rare. The *LAZY1* is the first identified gene-related regulation of the rice tiller angle. A mutation in *LAZY1* causes the rice tiller-spreading phenotype by modulating the endogenous IAA distribution in shoots [9]. Recently, the *prostrate growth1* variant, which encodes a single Cys2-His2 zinc-finger protein on chromosome 7, has been identified in rice and has been demonstrated to disrupt the *prostrate growth1* function and inactivate its expression, which leads to vertical growth and increases the grain number and yield of rice [10]. Moreover, genetic complementation further identified the *Tiller Angle Control 1* (*TAC*1) gene, which contributes significantly to rice yield and is generally utilized in rice breeding in Asia [11,12]. In addition, *OsHOX1* and *OsHOX28* are class II homeodomain-Leu zipper genes that are positive regulators of tiller angle by affecting shoot gravitropism [13]. Additionally, the *Tiller Angle Control 4* (*TAC4*) gene is a plant-specific and highly conserved nuclear protein that controls the tiller angle in rice. *TAC4* regulates rice geotropism by altering the auxin distribution and raising the concentration of indole acetic acid. When the *TAC4* function is lost, the tiller angle increases significantly [14]. Overall, these findings have improved our understanding of the tiller angle, although more research is required in this area. 

In the current study, we grew 120 doubled haploid lines derived from Cheongcheong (*Indica*) and Nagdong (*Japonica*) and collected data concerning tiller angle and tiller crown width for two years. The tiller angle and tiller crown width of the rice were then evaluated comprehensively using these data. The main purpose of this study is to use QTL analysis to detect the stable QTLs and identify the tiller angle-related genes from the target marker interval. Eight QTLs were detected in two years that affect the tiller angle and tiller crown width, the identification of which may lead to a better understanding of the genetic basis for tiller angle and tiller crown width. In particular, these QTLs offer a viable target for marker-assisted breeding in the development of ideal-type rice.

## 2. Results

### 2.1. Phenotype Evaluation for Tiller Angle and Tiller Crown Width in CNDH Population

**Figure 1:**
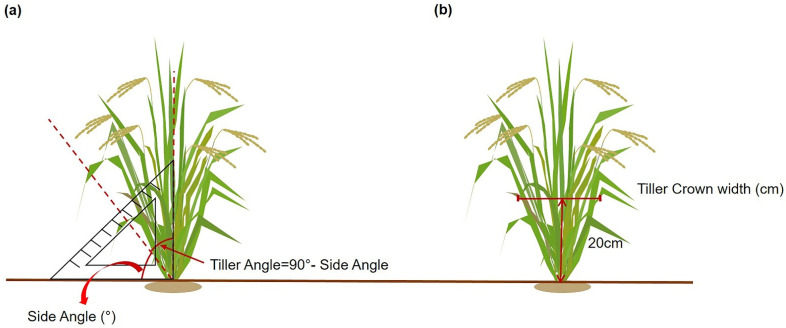
Method for measuring tiller angle and tiller crown width. (**a**) The side angle has measured the angle from the ground to the outermost tillers using a protractor. The tiller angle was calculated by subtracting the side angle from the 90° angle. (**b**) Method for measuring the tiller crown width. The width of the crown was determined by measuring the width of the tiller per plant at 20 cm above the soil surface.

**Figure 2:**
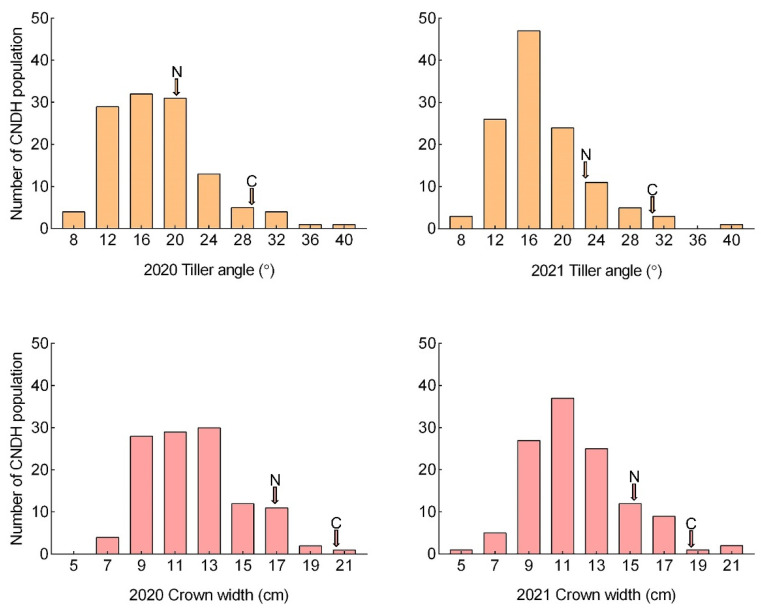
Frequency distribution of the tiller angle and tiller crown width in the CNDH population over two years for 2020 and 2021 (C, Cheongcheong; N, Nagdong). The tiller number and crown width show a normal distribution and are controlled by polygenes.

To perform the QTL analysis, the angle of the tiller and tiller crown width of the 120 CNDH (Cheongcheong/Nagdong doubled haploid) population and their parental lines of Cheongcheong and Nagdong were investigated in 2020 and 2021 (Figure 1). Based on comparative analyses of the tiller angle and tiller crown width gleaned from the two years’ data, it was observed that Cheongcheong was bigger than Nagdong (Table S1). In both years, the maximum tiller angle in the 120 CNDH population was 38° and the minimum was 8°. The average tiller angle in the CNDH population was around 17°. Moreover, using a histogram showed the CNDH population’s frequency distribution and the curves were similar to the normal distribution, which means that these plants’ traits are quantitative traits in which polygenes are controlled (Figure 2). In addition, the correlation analysis showed that the tiller angle and tiller crown width were positively correlated with each other (Table S2).

### 2.2. QTL Analysis Associated with Tiller Angle and Tiller Crown Width

**Figure 3:**
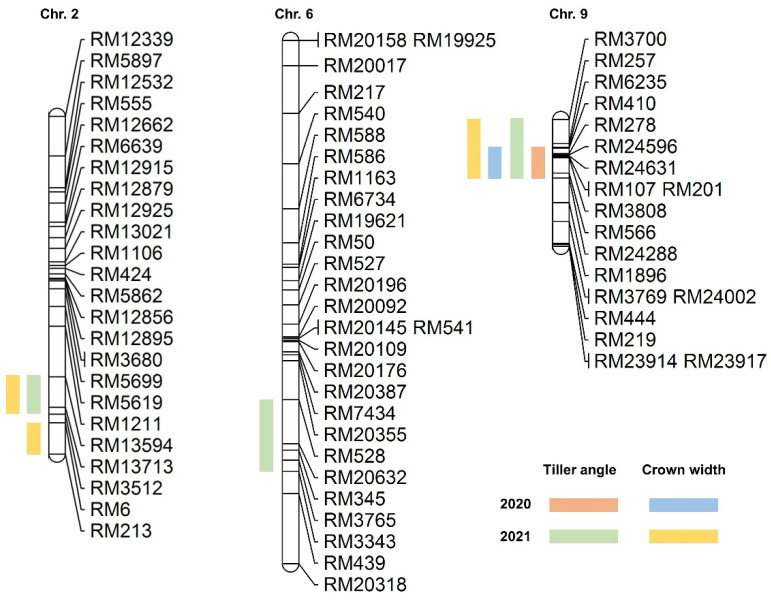
The location of QTL on chromosomes is associated with tiller angle and tiller crown width in the CNDH population. QTL analysis results show that tiller angle-related genes are located on chromosomes 2, 6, and 9. Among them, RM6235-RM24288 on chromosome 9 is a significant location that was detected over two years.

QTL analysis was performed using the tiller angle and tiller crown width of the 120 CNDH population (Table S3). In the QTL analysis, *qTA9* and *qCW9* on chromosome 9 were detected in 2020, and *qTA2* on chromosome 2, *qTA6* on chromosome 6, *qTA9-1* on chromosome 9, as well as *qCW2-1*, *qCW2-2*, and *qCW9-1*, were detected in 2021 (Table S3). In 2020, both *qTA9* and *qCW9* were located at RM6235–RM24288 on chromosome 9, with the LOD value being 4.27 and 5.66, respectively. The explainable phenotypic variations were 27% and 30%, respectively. Moreover, in 2021, *qTA2* and *qCW2-1* were detected in the same location on chromosome 2 at RM13594–RM3512. The LOD values were 4.26 and 3.21, respectively. The explainable phenotypic variation was 39% and 35%, respectively. *qTA6* was located at RM528–RM3343 on chromosome 6, along with a 3.12 LOD value, while the significant phenotypic variation was 37%. *qTA9-1* and *qCW9-1* were located at RM3700–RM24288 on chromosome 9 with 7.08 and 6.33 LOD values, respectively. The phenotypic variation that can be explained for both was 37%. In addition, *qCW2-2* was located at RM6–RM213 on chromosome 2 with 35% explainable phenotypic variation, and the LOD value was 3.76. In 2020 and 2021, all detected QTLs were derived from the Cheongcheong allele. Four QTLs (*qTA9*, *qCW9*, *qTA9-1*, and *qCW9-1*) were overlapped at marker interval RM6235–RM24288 on chromosome 9 with a large effect value found in 2020 and 2021. Therefore, tiller angle and tiller crown width within the interval RM6235–RM24288 on chromosome 9 were used to further screen for related genes (Figure 3).

### 2.3. Search for Related Genes Associated with Tiller Angle and Tiller Crown Width Based on QTL Analysis

**Figure 4:**
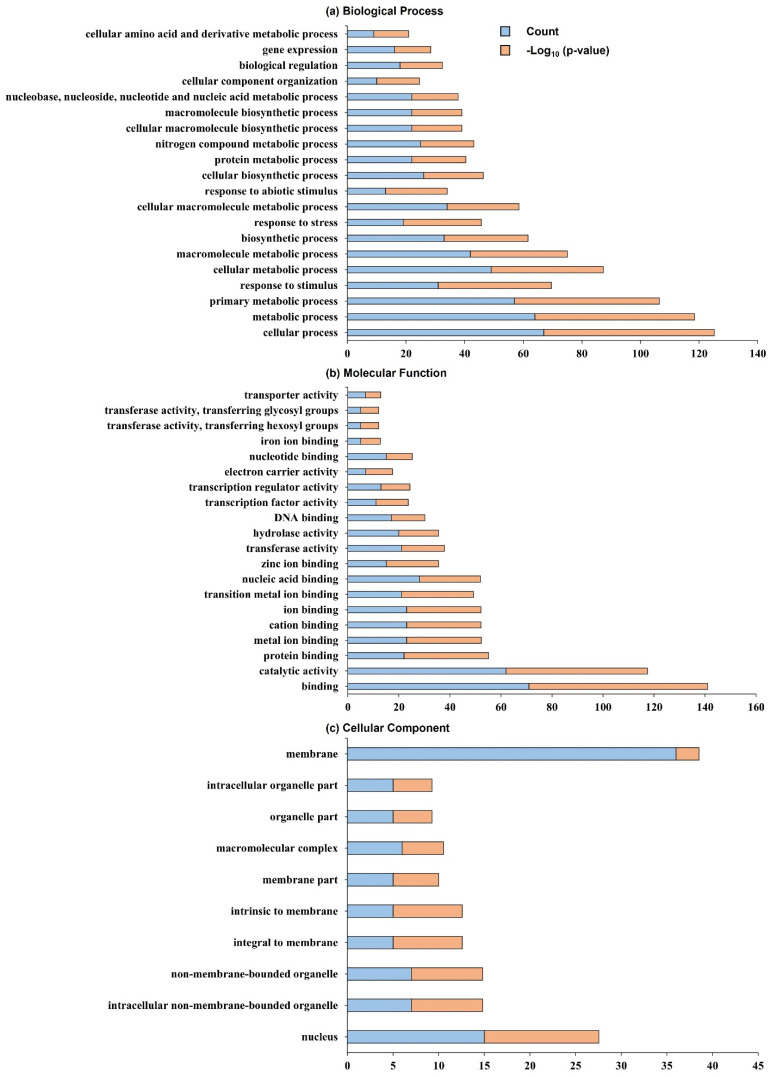
Gene ontology annotation of the QTL target locus RM6235–RM24288 on chromosome 9. (**a**) The 20 top-ranking GO terms for Biological Process. (**b**) The 20 top-ranking GO terms for Molecular Function. (**c**) The 10 top-ranking GO terms for Cellular Component.

In this research, QTL analysis was analyzed to detect target marker interval RM6235–RM24288 and to screen open reading frames (ORFs) related to the tiller angle and tiller crown width within this region. The RiceXpro database was used to search for tiller angle-related genes. Overall, 160 distinctive genes were identified in the target marker interval RM6235–RM24288. As a reference database, the AgriGo was used to summarize the rice gene identifiers of potential functional classifications. Within the target marker interval, RM6235–RM24288, significant GO terms for 160 individual genes were identified, and the 20, 20, and 10 highest significant terms were evaluated for “Biological Process,” “Molecular Function,” and “Cellular Component,”, respectively (Figure 4). Moreover, the highest significant GO terms related to “Biological Process’’ include cellular amino acid and its derivative metabolic process, biological regulation, gene expression, and cellular component organization; the terms most frequently related to “Molecular Function” include transferase and transporter activity, transferring glycosyl groups, transferring hexosyl groups, iron ion binding, and nucleotide-binding; and finally, the most significant GO terms for “Cellular Component” included membrane, intracellular organelle part, organelle part, macromolecular complex, and membrane part. Moreover, a total of 20 associated genes in the area of RM6235–RM24288 were selected depending on the sequence annotations that were available (Table S4).

### 2.4. Selection of Related Genes and Comparison of Relative Expression Level of Other Tiller Angle-Related Genes

**Figure 5:**
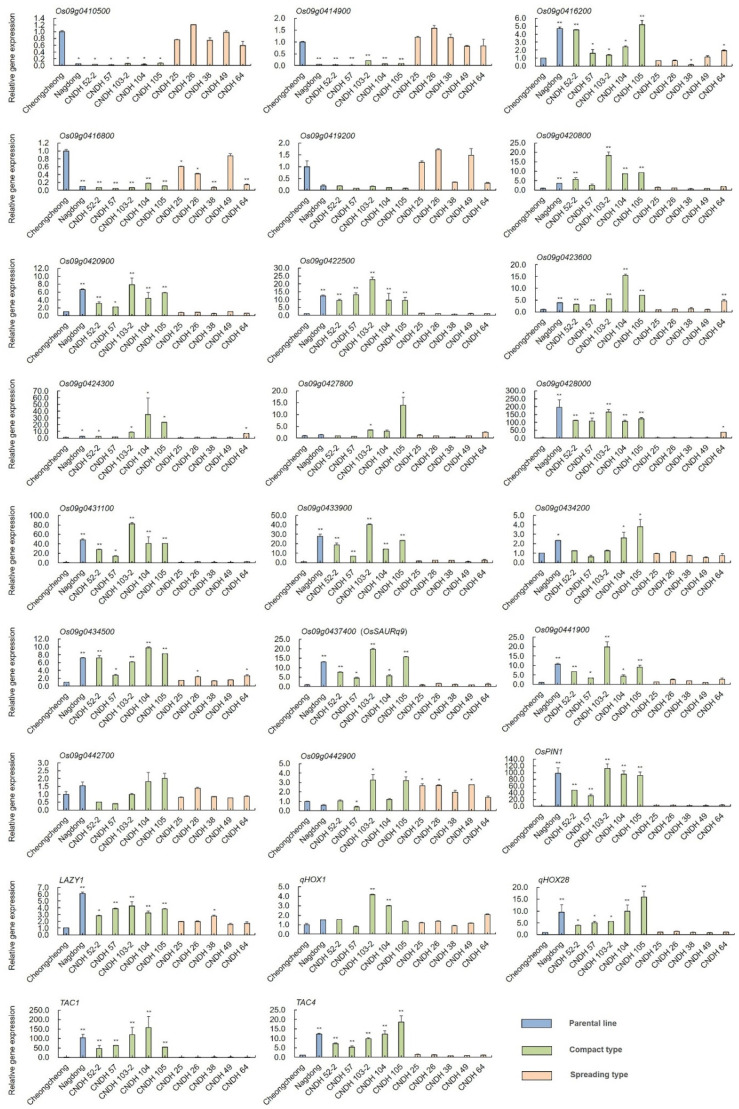
Comparison between the 20 selected tiller angle-related genes by QTL analysis and the relative expression levels of six tiller angle-related genes that have already been reported. The expression of tiller angle-related genes in Cheongcheong, Nagdong, compact type (CNDH 52-2, CNDH 57, CNDH 103-2, CNDH 104, and CNDH 105), and spreading type (CNDH 25, CNDH 26, CNDH 38, CNDH49, and CNDH64). Values are the means ± SD (n = 3). Stars on each bar represent the significant difference at (*) *p* < 0.05, and (**) *p* < 0.01 (two-tailed Student’s *t*-test).

**Figure 6:**
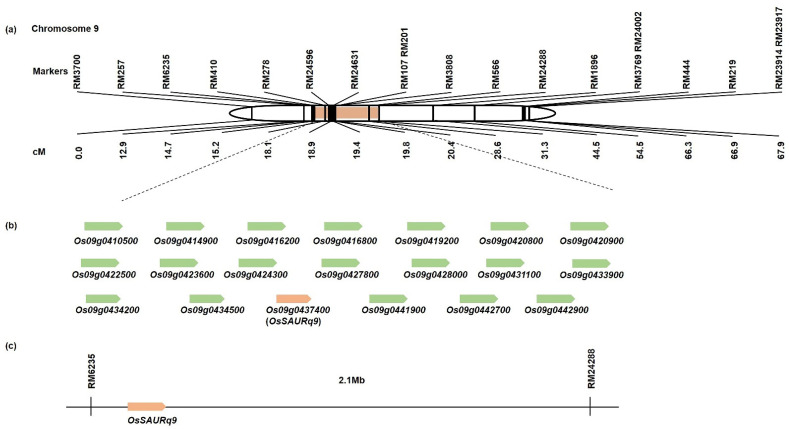
Physical mapping of the genes associated with tiller angle and tiller crown width. (**a**) Target marker interval RM6235–RM24288 on chromosome 9 that overlaps two years for tiller angle and tiller crown width. (**b**) Twenty representative related genes were predicted in the 2.1 Mb region between marker RM6235 and RM24288. (**c**) *OsSAURq9* has finally been select as a target preferred gene related to rice tiller angle.

The relative expression of genes associated with tiller angle and tiller crown width were analyzed at the heading stage. Twenty relative genes were selected from the target region RM6235–RM24288 chromosome 9, and we also measured six other tiller angle-related genes, including *OsPIN1*, *LAZY1*, *qHOX1*, *qHOX28*, *TAC1*, and *TAC4* (Figure 5). These genes are representative tiller angle control genes that have been reported in previous studies [14,15,16,17,18]. In the 120 CNDH population, five compact type lines (CNDH 52-2, CNDH 57, CNDH 103-2, CNDH 104, and CNDH 105), five spreading type lines (CNDH 25, CNDH 26, CNDH 38, CNDH 49, and CNDH 64), and the two parental lines (Cheongcheong and Nagdong) were selected. The relative expression level of *Os09g0410500*, *Os09g0414900*, and *Os09g0416800* was significantly different between Cheongcheong and Nagdong, with Cheongcheong being higher than Nagdong. Among these three genes, compact type lines are significantly different from Cheongcheong as well as spreading type lines, except in *Os09g0416800*. The expression levels of *Os09g0419200*, *Os09g0427800*, *Os09g0442700*, *Os09g0442900*, and *qHOX1* were not significantly different between Cheongcheong and Nagdong. The relative expression levels of *Os09g0416200*, *Os09g0420800*, *Os09g0420900*, *Os09g0422500*, *Os09g0423600*, *Os09g0424300*, *Os09g0428000*, *Os09g0431100*, *Os09g0433900*, *Os09g0434200*, *Os09g0434500*, *Os09g0437400*, *Os09g0441900*, *OsPIN1*, *OsLAZY1*, *qHOX28*, *TAC1*, and *TAC4* were significantly different between Cheongcheong and Nagdong, with Cheongcheong’s expression levels of these genes being lower than those of Nagdong. Among them, six genes (*Os09g0420900*, *Os09g0422500*, *Os09g0431100*, *Os09g0433900*, *Os09g0437400*, and *Os09g0441900*) from the target region RM6235–RM24288 were found to have remarkably higher levels in the compact type lines than in the Cheongcheong or spreading type lines. Among them, *Os09g0437400* (*OsSAURq9*) was selected as the preferred gene in this study, which belongs to SAUR—an auxin-responsive protein family (Figure 6).

### 2.5. Phylogenetic Tree and Homology Sequence Analysis of OsSAURq9

**Figure 7:**
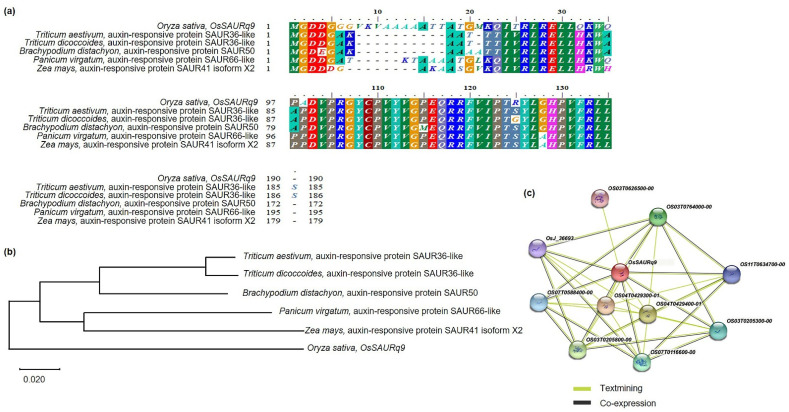
Sequence analysis of *OsSAURq9*. (**a**) The high similarity protein sequences with *OsSAURq9* were found in *Triticum aestivum*, *Triticum dicoccoides*, *Brachypodium distachyon*, *Panicum virgatum*, and *Zea mays*. (**b**) The gene and homologous gene were analyzed using a phylogenetic tree using 1000 bootstrap replicates to build the phylogenetic tree through the parsimony method. (**c**) Protein interactions of *OsSAURq9*. *OsSAURq9* interacts with ten different proteins (OS04T0429300-01, OS04T0429400-01, OS03T0205800-00, OS03T0764000-00, OS07T0116600-00, OS03T0205300-00, OS07T0588400-00, OS11T0634700-00, OsJ_36693, and OS03T0626500-00).

The preferred gene *OsSAURq9*, which is associated with tiller angle, was finally screened by QTL analysis and relative expression level analysis. Furthermore, the results of BLAST analysis by the NCBI database indicate that *OsSAURq9* had a very similar sequence to that of auxin-responsive protein SAUR36 in *Triticum aestivum* and *Triticum dicoccoides*, auxin-responsive protein SAUR50 in *Brachypodium distachyon*, auxin-responsive protein SAUR66 in *Panicum virgatum*, and auxin-responsive protein SAUR41 isoform X2 in *Zea mays* (Figure 7a). The phylogenetic tree analysis was used for clarifying the genetic similarities between the *OsSAURq9* of *Triticum aestivum*, *Triticum dicoccoides*, *Brachypodium distachyon*, *Panicum virgatum*, and *Zea mays* (Figure 7b). Moreover, the *OsSAURq9* domain was used to reveal the function of the partners; it was observed that *OsSAURq9* shows interactions with ten different proteins (OsJ-36693, OS07T0588400-00, OS03T0205800-00, OS07T0116600-00, OS04T0429300-01, OS04T0429400-01, OS03T0205300-00, OS11T0634700-00, OS03T0764000-00, and OS03T0626500-00) (Figure 7c).

## 3. Discussion

The tiller angle is determined through QTLs generated from natural variations in rice [12,19,20]. Therefore, the dissection of key genetic variations to control rice tiller angle is important for breeding as they are potential sources of important alleles. QTL analysis is a useful method for discovering beneficial alleles in natural resources [21]. In the current study, QTL analysis was used for a 120 doubled haploid population from a cross between a spreading type *Indica* variety “Cheongcheong” and a compact type *Japonica* variety “Nagdong”. QTL analysis of tiller angle and tiller crown width were used to detect target loci that play a vital role in these traits. Most of the previous reports only focused on the tiller angle for rice architecture [4,7]. However, in this study, we also measured the tiller crown width for a more accurate analysis. The results analyzed the tiller angle and tiller crown width, which are quantitative traits that exhibit continuous variation and follow a normal distribution. This means that the tiller angle and tiller crown width are encoded by the interaction of polygenes [22]. Moreover, the correlation analysis between tiller angle and tiller crown width showed a positive correlation with each other. We examined the accuracy of the field investigation for tiller angle and tiller crown width. Based on two years of QTL analysis, in 2020, both *qTA9* and *qCW9* were located at RM6235–RM24288 on chromosome 9; however, in 2021, more QTLs were detected on chromosomes 2, 6, and 9. Environmental factors might have influenced the difference between the two years [23]. In addition, four QTLs (*qTA9*, *qTA9-1*, *qCW9*, and *qCW9-1*) overlapped in the same marker interval of RM6235–RM24288 on chromosome 9 with the highest LOD value of 7.08. Therefore, RM6235–RM24288 is the target location to further screen related genes for tiller angle and tiller crown width. Furthermore, 160 tiller angle-related genes were screened, and from among them, twenty genes were selected by gene function annotation. Moreover, to search for a more suitable and closely correlated gene that could play a more vital role in tiller angle, the relative expression level of 20 related genes and 6 reported tiller angle control genes were analyzed. Five compact type lines (CNDH 52-2, CNDH 57, CNDH 103-2, CNDH 104, and CNDH 105), and five spreading type lines (CNDH 25, CNDH 26, CNDH 38, CNDH49, and CNDH64) from 120 CNDH populations with the parental lines Cheongcheong and Nagdong were selected to compare gene expression. The results indicated that the expression levels of six genes (*Os09g0420900*, *Os09g0422500*, *Os09g0431100*, *Os09g0433900*, *Os09g0437400*, and *Os09g0441900*) selected from the target marker interval RM6235–RM24288 and four genes (*OsPIN1*, *qHOX28*, *TAC1*, and *TAC4*) that have already been reported to be involved in tiller angle were significantly different between Cheongcheong and Nagdong. Their expression levels were significantly higher in Nagdong and compact type lines than in Cheongcheong and spreading type lines. Therefore, *Os09g0437400* (*OsSAURq9*) was finally selected as a preferred gene for further analysis. *OsSAURq9* is an auxin-responsive SAUR protein.

*OsSAURq9* has a very similar sequence to auxin-responsive SAUR proteins in *Triticum aestivum*, *Triticum dicoccoides*, *Brachypodium distachyon*, *Panicum virgatum*, and *Zea mays*. *SAUR* is an early auxin-responsive gene [24]. The *SAUR* family genes are associated with auxin biosynthesis, signaling, and the response of rice shoots to gravity [17]. For example, transgenic plants overexpressing the *SAUR39* gene in rice exhibit the reduced growth of shoot and root and increased the angle of leaf and tiller compared to wild-type plants [25]. Similarly, *OsSAUR45* is involved in rice growth and development and inhibits the expression of *OsYUCCA* and *OsPIN* genes, thereby affecting the synthesis and transport of auxin [26]. The asymmetrical distribution of auxin plays an important role in plant gravity response and the regulation of the tiller angle [14]. The *LAZY1* was shown to regulate the tiller angle of rice by asymmetrical auxin distribution caused by polar auxin transport [9,27]. Previous studies have shown that auxin and its signal transduction may be the key regulatory factors of tiller development [28]. Rice develops tillers from axillary buds, and transgenic plants with reduced expression of the *OsPIN1* gene have higher tiller numbers and tiller angles [15]. Genes associated with auxin transport play a key role in regulating tiller angle [29]. 

In the present study, *OsSAURq9* has finally been selected from the twenty tiller angle-related genes in the target marker interval RM6235–RM24288. Additionally, for future investigation, the identification of these genes will be valuable to understanding the mechanisms involved in determining the tiller angle. Our findings concerning QTLs and related genes can provide a unique genetic resource for further gene discovery and breeding related to the rice tiller angle.

## 4. Materials and Methods

### 4.1. Plant Material and Experiment Design

In the current study, a doubled haploid population consisting of 120 lines by anther culture using an F_1_ population derived by a cross between *O. sativa spp. Indica* Cheongcheong and *O. sativa spp. Japonica* Nagdong was used [30]. The CNDH population was used as a bridge parent for more than 10 years and was grown in a paddy field at Gunwi-gun, Gyeongbuk, Republic of Korea (36°11′ N, 128°64′ E). The present experiments were conducted from April to October in 2020 and 2021. All plants were grown in the test field, Kyungpook National University, Gunwi, Republic of Korea. The 120 CNDH population along with their parents Cheongcheong/Nagdong were planted as 122 plots. All the plots were randomized into a block design. A 25% prochloraz (Hankook Samgong, Seoul, Korea) was used to sterilize the seeds and soaked at 33 °C for three days under dark conditions using an incubator before sowing. Thirty days after sowing, seedlings were planted into the fields under the following conditions: one seedling per hill, 24 seedlings per row, with a density of 0.15 m × 0.3 m, and six rows per accession. All the field management followed international guidelines, and the methods of the Rural Development Administration regarding insecticides and herbicides were followed to control pests and diseases. The standard fertilization amounts of N, K_2_O, and P_2_O_5_ were used (9, 5.7, and 4.5 kg per 10 ha, respectively). 

### 4.2. Measurement of Tiller Angle and Tiller Crown Width

The angle of the tiller is described as the angle of the main stem with the outside tillers at maximum inclination [3]. A protractor was used to measure the angle between the outermost side tiller and the horizontal ground as a side angle at the heading stage [31]. The tiller angle was calculated by subtracting the side angle from 90° [32]. The measurement method is shown in Figure 1. The width of the tiller crown was determined as the width of tillers per plant at 20 cm above the soil surface [33]. An average of six plants were used in the QTL analysis.

### 4.3. Mapping Population and QTL Analysis for Tiller Angle and Tiller Crown Width

To construct the CNDH population map, the 788 SSR markers were used. The polymorphism analysis detected 423 SSR markers among them. Finally, 222 SSR markers were chosen from co-dominant genes that were amplified via PCR amplification [30]. The genetic map of the CNDH population has an average distance of 10.6 cM between markers and includes a total of 2121.7 cM genetic distance [34]. The CNDH genetic map was created using Mapmarker version 3.0 and the markers were distributed evenly across the 12 chromosomes of rice [35]. The QTL analysis of the tiller angle and tiller crown width was carried out with Windows QTL cartographer 2.5 [36]. A composite interval mapping was used to scan the whole-genome QTLs. The LOD score was set at 3.0 or higher to enhance the accuracy of the detected QTL. The required data for the QTL analysis include the number of chromosomes, the genetic distance of the marker, genotyping data, and the values of target plant traits entered. The naming of the detected QTL depends on the nomenclature reported by McCough and Doerge [37].

### 4.4. Prediction of the Related Genes

Based on QTL mapping results, the genes related to the tiller angle and tiller crown width were screened using Rapdb (https://rapdb.dna.affrc.go.jp/ (accessed on 10 April 2022) and RiceXpro (https://ricexpro.dna.affrc.go.jp/ (accessed on 10 April 2022)) [38]. Rapdb and RiceXpro selected all the ORFs in the target QTL region, and the agriGO tool (http://bioinfo.cau.edu.cn/agriGO/ (accessed on 10 April 2022)) was applied to identify the function of all related genes by the gene ontology (GO) enrichment analysis [39]. Based on the classification of gene functions, we searched for genes that influence tiller angle and tiller crown width. For the multiple homologous sequence variation analyses of the related genes comparisons, NCBI (National Center for Biotechnology Information, Bethesda, Maryland, USA, https://www.ncbi.nlm.nih.gov/ (accessed on 10 April 2022)) and BioEdit 7.0 (https://bioedit.software.informer.com/7.0/ (accessed on 10 April 2022)) were used [40]. Moreover, the MEGA 11 (https://www.megasoftware.net/ (accessed on 10 April 2022)) software was used to erect the phylogenetic tree. A STRING (version 11.0) database (https://string-db.org/ (accessed on 10 April 2022)) was used to analyze the protein–protein interaction/association network [41].

### 4.5. Expression Level of Related Genes

To find out whether the related genes detected by QTL analysis influence tiller angle and tiller crown width, the expression levels of related genes were checked. Among the CNDH population, five compact type lines (CNDH 52-2, CNDH 57, CNDH 103-2, CNDH 104, and CNDH 105), five spreading type lines (CNDH 25, CNDH 26, CNDH 38, CNDH 49, and CNDH 64), and Cheongcheong and Nagdong (parental lines) were selected. At the heading stage, rice leaves were sampled. For total RNA extraction, the RNeasy Mini Kit (QIAGEN, Hilden, Germany) was used following the manufacturer’s instructions. To obtain a 100 ng/µL concentration, the RNA was further diluted using Rnase-free water, and cDNA was produced using the UltraScript 2.0 cDNA Synthesis Kit with 100 ng of RNA as a template (PCR Biosystems, cat. No. PB30.31.10, Wayne, Pennsylvania, USA). The obtained complementary DNA was utilized as a template in the StepOnePlusTM RT-PCR System machine (Thermo Fisher Scientific, Seoul, Korea) with the 2X Real-time PCR Master Mix (containing SYBR^®^ Green I, cat. No. DQ383-40h, BioFACTTM, Daejeon, Korea) and 10 nM of each primer as well as 100 ng of template DNA to a total volume of 20 µL. A 2-step PCR reaction for 40 cycles was applied under the following setup: polymerase activation at 95 °C for 15 min, denaturation and annealing at 95 °C for 20 s, and extension at 60 °C for 40 s. For the internal reference gene, *OsActin* was used. To calculate the average and standard deviation, each reaction was carried out with three replicates, and the list of primers used in this investigation is provided in Table S5.

### 4.6. Statistical Analysis

The average measurements of six plants for the tiller angle and tiller crown width were used for subsequent analyses. GraphPad Prism was used to create and analyze the frequency distribution graph (version 8.0.2, Dotmatics, San Diego, CA, USA). SPSS statistical software (version 26, IBM, New York, NY, USA) was used for the Pearson’s correlation analysis. The significance of the difference was determined using one-way variance analysis and the Student’s *t*-test.

## 5. Conclusions

We performed QTL analysis to examine the tiller angle and tiller crown width of rice plants for two consecutive years. Our results showed that four QTLs overlap at the target marker interval RM6235–RM24288 on chromosome 9. Twenty tiller angle-related genes were selected from the target region and the relative gene expression levels were checked in five compact type lines, five spreading type lines, and their parental lines. Of these, *OsSAURq9*, which belongs to an auxin-responsive SAUR protein family, was chosen as a preferred target gene. Further investigation is required to clarify the molecular mechanisms of rice. *OsSAURq9* was newly discovered in this study and may be useful in further research on tiller angle to aid in the development of ideal-type rice.

## Data Availability

The data presented in this study are available on request from the corresponding author.

## Supplementary Materials

The following supporting information can be downloaded at: https://www.mdpi.com/article/10.3390/ijms23095192/s1.

## References

[1] Wang Y., Li J. (2006). Genes controlling plant architecture. Curr. Opin. Biotechnol..

[2] Li Y., Li J., Chen Z., Wei Y., Qi Y., Wu C. (2020). OsmiR167a-targeted auxin response factors modulate tiller angle via fine-tuning auxin distribution in rice. Plant Biotechnol. J..

[3] Dong H., Zhao H., Xie W., Han Z., Li G., Yao W., Bai X., Hu Y., Guo Z., Lu K. (2016). A Novel Tiller Angle Gene, TAC3, together with TAC1 and D2 Largely Determine the Natural Variation of Tiller Angle in Rice Cultivars. PLoS Genet..

[4] Chen P., Jiang L., Yu C., Zhang W., Wang J., Wan J. (2008). The Identification and Mapping of a Tiller Angle QTL on Rice Chromosome 9. Crop Sci..

[5] Xu Y., McCouch S.R., Shen Z. (1998). Transgressive Segregation of Tiller Angle in Rice Caused by Complementary Gene Action. Crop Sci..

[6] Wang Y., Li J. (2005). The Plant Architecture of Rice (*Oryza sativa*). Plant Mol. Biol..

[7] He J., Shao G., Wei X., Huang F., Sheng Z., Tang S., Hu P. (2017). Fine mapping and candidate gene analysis of qTAC8, a major quantitative trait locus controlling tiller angle in rice (*Oryza sativa* L.). PLoS ONE.

[8] Li Z., Paterson A.H., Pinson S.R.M., Stansel J.W. (1999). RFLP facilitated analysis of tiller and leaf angles in rice (*Oryza sativa* L.). Euphytica.

[9] Yoshihara T., Iino M. (2007). Identification of the Gravitropism-Related Rice Gene LAZY1 and Elucidation of LAZY1-Dependent and -Independent Gravity Signaling Pathways. Plant Cell Physiol..

[10] Tan L., Li X., Liu F., Sun X., Li C., Zhu Z., Fu Y., Cai H., Wang X., Xie D. (2008). Control of a key transition from prostrate to erect growth in rice domestication. Nat. Genet..

[11] Yu B., Lin Z., Li H., Li X., Li J., Wang Y., Zhang X., Zhu Z., Zhai W., Wang X. (2007). TAC1, a major quantitative trait locus controlling tiller angle in rice. Plant J..

[12] Jiang J., Tan L., Zhu Z., Fu Y., Liu F., Cai H., Sun C. (2012). Molecular Evolution of the TAC1 Gene from Rice (*Oryza sativa* L.). J. Genet. Genom..

[13] Hu Y., Li S., Fan X., Song S., Zhou X., Weng X., Xiao J., Li X., Xiong L., You A. (2020). OsHOX1 and OsHOX28 Redundantly Shape Rice Tiller Angle by Reducing HSFA2D Expression and Auxin Content. Plant Physiol..

[14] Li H., Sun H., Jiang J., Sun X., Tan L., Sun C. (2021). TAC4 controls tiller angle by regulating the endogenous auxin content and distribution in rice. Plant Biotechnol. J..

[15] Xu M., Zhu L., Shou H., Wu P. (2005). A PIN1 Family Gene, OsPIN1, involved in Auxin-dependent Adventitious Root Emergence and Tillering in Rice. Plant Cell Physiol..

[16] He Y., Li L., Jiang D. (2021). Understanding the Regulatory Mechanisms of Rice Tiller Angle, Then and Now. Plant Mol. Biol. Report..

[17] Zhang N., Yu H., Yu H., Cai Y., Huang L., Xu C., Xiong G., Meng X., Wang J., Chen H. (2018). A Core Regulatory Pathway Controlling Rice Tiller Angle Mediated by the LAZY1-Dependent Asymmetric Distribution of Auxin. Plant Cell.

[18] Waite J.M., Dardick C. (2018). TILLER ANGLE CONTROL 1 modulates plant architecture in response to photosynthetic signals. J. Exp. Bot..

[19] Zhang W., Tan L., Sun H., Zhao X., Liu F., Cai H., Fu Y., Sun X., Gu P., Zhu Z. (2019). Natural Variations at TIG1 Encoding a TCP Transcription Factor Contribute to Plant Architecture Domestication in Rice. Mol. Plant.

[20] Jin J., Huang W., Gao J.-P., Yang J., Shi M., Zhu M.-Z., Luo D., Lin H.-X. (2008). Genetic control of rice plant architecture under domestication. Nat. Genet..

[21] Wang Y., Li J. (2008). Rice, rising. Nat. Genet..

[22] Xu S. (2013). Mapping Quantitative Trait Loci by Controlling Polygenic Background Effects. Genetics.

[23] Price A., Courtois B. (1999). Mapping QTLs associated with drought resistance in rice: Progress, problems and prospects. Plant Growth Regul..

[24] Hagen G., Guilfoyle T. (2002). Auxin-responsive gene expression: Genes, promoters and regulatory factors. Plant Mol. Biol..

[25] Kant S., Bi Y.-M., Zhu T., Rothstein S.J. (2009). SAUR39, a Small Auxin-Up RNA Gene, Acts as a Negative Regulator of Auxin Synthesis and Transport in Rice. Plant Physiol..

[26] Xu Y.-X., Xiao M.-Z., Liu Y., Fu J.-L., He Y., Jiang D.-A. (2017). The small auxin-up RNA OsSAUR45 affects auxin synthesis and transport in rice. Plant Mol. Biol..

[27] Li P., Wang Y., Qian Q., Fu Z., Wang M., Zeng D., Li B., Wang X., Li J. (2007). LAZY1 controls rice shoot gravitropism through regulating polar auxin transport. Cell Res..

[28] Song Y., Xu Z.-F. (2013). Ectopic Overexpression of an AUXIN/INDOLE-3-ACETIC ACID (Aux/IAA) Gene OsIAA4 in Rice Induces Morphological Changes and Reduces Responsiveness to Auxin. Int. J. Mol. Sci..

[29] Shen Y., Yang Y., Xu E., Ge X., Xiang Y., Li Z. (2018). Novel and major QTL for branch angle detected by using DH population from an exotic introgression in rapeseed (*Brassica napus* L.). Theor. Appl. Genet..

[30] Yun B.-W., Kim M.-G., Handoyo T., Kim K.-M. (2014). Analysis of rice grain quality-associated quantitative trait loci by using genetic mapping. Am. J. Plant Sci..

[31] Wang W., Gao H., Liang Y., Li J., Wang Y. (2022). Molecular basis underlying rice tiller angle: Current progress and future perspectives. Mol. Plant.

[32] Jang S., Kang Y.S., Lee Y.K., Koh H.-J. (2020). Evaluating Multiple Allelic Combination to Determine Tiller Angle Variation in Rice. Agriculture.

[33] Kashiwagi T., Togawa E., Hirotsu N., Ishimaru K. (2008). Improvement of lodging resistance with QTLs for stem diameter in rice (*Oryza sativa* L.). Theor. Appl. Genet..

[34] Kim T., Kim K., Manigbas N.L., Yi G., Sohn J. (2013). Identification of quantitative trait loci for resistance to white-backed planthopper (*Sogatella furcifera*) in rice with Milyang 46 (Cheongcheongbyeo) background. Philipp. J. Crop Sci..

[35] Lincoln S.E., Daly M.J., Lander E.S. (1993). Constructing Genetic Linkage Maps with MAPMAKER/EXP Version 3.0: A Tutorial and Reference Manual. A Whitehead Institute for Biomedical Research Technical Report.

[36] Zeng Z.B. (1994). Precision mapping of quantitative trait loci. Genetics.

[37] McCough S.R., Doerge R.W. (1995). QTL mapping in rice. Trends Genet..

[38] Sato Y., Takehisa H., Kamatsuki K., Minami H., Namiki N., Ikawa H., Ohyanagi H., Sugimoto K., Antonio B.A., Nagamura Y. (2013). RiceXPro Version 3.0: Expanding the informatics resource for rice transcriptome. Nucleic Acids Res..

[39] Du Z., Zhou X., Ling Y., Zhang Z., Su Z. (2010). agriGO: A GO analysis toolkit for the agricultural community. Nucleic Acids Res..

[40] Hall T. (2004). BioEdit.

[41] Szklarczyk D., Gable A.L., Lyon D., Junge A., Wyder S., Huerta-Cepas J., Simonovic M., Doncheva N.T., Morris J.H., Bork P. (2019). STRING v11: Protein–protein association networks with increased coverage, supporting functional discovery in genome-wide experimental datasets. Nucleic Acids Res..

