# Supplementary material for: Identification of a Major QTL and Validation of Related Genes for Tiller Angle in Rice Based on QTL Analysis
Source: Int J Mol Sci. 2022 May 6;23(9):5192. doi: 10.3390/ijms23095192 (PMC9105483; doi:10.3390/ijms23095192)
Supplement: Supplementary file 1 [file ijms-23-05192-s001.zip › ijms-1702649-supplementary.pdf]

Table S1. Phenotypic values of tiller angle and tiller crown width in the CNDH population and its parents, "Cheongcheong" and "Nagdong".

Trait	Year	Parents		DH Population		
		Cheongcheong	Nagdong	Max.	Min.	Mean
Tiller angle (°)	2020	30.0±0.8	21.0±1.0	38.0	8.0	17.4±5.7
	2021	31.0±2.1	23.0±1.7	38.0	8.0	17.1±5.2
Crown width (cm)	2020	20.5±0.8	17.0±1.3	26.0	6.8	12.3±3.3
	2021	18.5±1.3	15.5±0.9	22.0	5.8	11.8±3.0

Table S2. The correlation of tiller angle and tiller crown width from the 120 CNDH populations in 2020 and 2021.

Year	Plant Trait	Tiller Angle	Crown Width	Tiller Angle	Crown Width
2020	Tiller angle	1.000			
	Crown width	0.794**	1.000		
2021	Tiller angle	0.691**	0.682**	1.000	
	Crown width	0.705**	0.790**	0.820**	1.000

** Significant at 0.01 level.

Table S3. QTLs related to the tiller angle and tiller crown width of the CNDH population in two consecutive years.

Trait	Year	Chromosome	QTL	Marker Interval [a]	LOD	Add. Effect [b]	R^2 [c]	Increasing Effect [d]
Tiller angle	2020	9	*qTA9*	RM6235-RM24288	4.27	2.44	0.27	Cheongcheong
	2021	2	*qTA2*	RM13594-RM3512	4.26	1.98	0.39	Cheongcheong
		6	*qTA6*	RM528-RM3343	3.12	1.60	0.37	Cheongcheong
		9	*qTA9-1*	RM3700-RM24288	7.08	2.52	0.37	Cheongcheong
Crown width	2020	9	*qCW9*	RM6235-RM24288	5.66	1.65	0.30	Cheongcheong
	2021	2-1	*qCW2-1*	RM13594-RM3512	3.21	1.10	0.35	Cheongcheong
		2-2	*qCW2-2*	RM6-RM213	3.76	1.08	0.35	Cheongcheong
		9	*qCW9-1*	RM3700-RM24288	6.33	1.43	0.37	Cheongcheong

[a] Marker Interval are those within the significance threshold on each border of the QTL range. [b] Additive effect. [c] Phenotypic variation explains each QTL. [d] Increase effect is the source of the allele causing an increase in the measured traits.

Table S4. Genes related to the tiller angle from the target interval RM6235–RN24288 on chromosome 9.

RAP-ID	MSU-ID	Start..End	Description
Os09g0410500	LOC_Os09g24480	14566850..14568219	Similar to SfCYC2 protein (Fragment).
Os09g0414900	LOC_Os09g24840	14828981..14830515	Similar to GASA5-like protein (Fragment).
Os09g0416200	LOC_Os09g24924	14887140..14893226	Similar to Glucose transporter (Fragment).
Os09g0416800	LOC_Os09g24990	14929157..14930268	Similar to CCR4-NOT transcription complex subunit 7 (CCR4-associated factor 1) (CAF1) (BTG1 binding factor 1).
Os09g0419200	LOC_Os09g25150	15076258..15079326	NAD-dependent epimerase/dehydratase family protein.
Os09g0420800	LOC_Os09g25320	15160893..15162859	Similar to Ubiquitin.
Os09g0420900	LOC_Os09g25330	15163139..15168798	BTB domain containing protein.
Os09g0422500	LOC_Os09g25490	15281320..15285893	Similar to Cellulose synthase (Fragment).
Os09g0423600	LOC_Os09g25580	15349074..15355640	Similar to Monogalactosyldiacylglycerol synthase (EC 2.4.1.46).
Os09g0424300	LOC_Os09g25620	15385723..15389096	S-adenosylmethionine decarboxylase.
Os09g0427800	LOC_Os09g25890	15532892..15535767	Glycosyl transferase, family 20 domain containing protein.
Os09g0428000	LOC_Os09g25900	15546372..15548565	Glycosyl transferase, family 2 domain containing protein.
Os09g0431100	LOC_Os09g26144	15752949..15762817	GPCR, family 3, metabotropic glutamate receptor-like protein.
Os09g0433900	LOC_Os09g26380	15930510..15936552	Similar to Alanine aminotransferase 2 (EC 2.6.1.2) (GPT) (Glutamic--pyruvic transaminase 2) (Glutamic--alanine transaminase 2) (ALAAT-2).
Os09g0434200	LOC_Os09g26400	15944563..15947917	Zinc finger, RING-type domain containing protein.
Os09g0434500	LOC_Os09g26420	15959434..15962646	Similar to Ethylene response factor 2.
Os09g0437400 (*OsSAURq9*)	LOC_Os09g26610	16143938..16144535	Auxin responsive SAUR protein family protein.
Os09g0441900	LOC_Os09g26999	16411151..16415851	Whey acidic protein, core region domain containing protein.
Os09g0442700	LOC_Os09g27060	16461644..16467575	SNF2-related domain containing protein.
Os09g0442900	LOC_Os09g27080	16477066..16481692	Similar to Axi 1 (Auxin-independent growth promoter)-like protein.

Table S5. List of primers used in this study.

Name	Forward Primer (5′-3′)	Reverse Primer (5′-3′)
Os09g0410500	CAGCAGCATCAGTACGACCA	GTTTCTTGCCGCCGTCAG
Os09g0414900	CATTGCATGGTTTGTCCAAG	GTTGTTGTAGCAGGGGCACT
Os09g0416200	GCGGTGAACCTCTTCTTCAC	TCCATAGGAGCACCATCTCC
Os09g0416800	ACCTTCTCGAATGAGCATGG	GTAACCCAGCGAACCTCAGA
Os09g0419200	AAGCTCTTCCCCGAGTATCC	AGGCTCTTCACCGTCTCGTA
Os09g0420800	TTTGTTCACTGTCGCAGGAC	TTCTCCTCAACACGCTCCTT
Os09g0420900	CTTGGTCCGAAGAGCTGAAG	TCTCCCCTTGGATTTGACAG
Os09g0422500	CCGTCGAGATCTTCTTCAGC	CAAACTCGCAAACGTGCTAA
Os09g0423600	AGTCCTTGGTGAACCCACTG	ATGGCCTCTGCAATTGTACC
Os09g0424300	TGAGCTTGCTGCAGAGTTGT	GAGTGTAACCATGGGCTGCT
Os09g0427800	GCATCCGTGTGCATTAGAGA	GTCTCCTTCGCCTCGATGTA
Os09g0428000	CTGTGCCTTCCGGACATACT	GCTCAGCCTCAGGAACAAAC
Os09g0431100	CCTCAAGTCCTACTGCGACA	GCTCGATGAGGTTCATCTCC
Os09g0433900	TCAATTTCTTTCGCGAGGTT	TCCATCAGCAACTGCTTCAC
Os09g0434200	GGCTTGTTGCCCAGAATTTA	TCCATCCACTGTCATGGTGT
Os09g0434500	GCAAGAAAGCCAAGGTCAAC	CGAATGTCTCAGTGGAAGCA
Os09g0437400 (OsSAURq9)	GAGGAAGACGAGGAGGTGGT	AGGTACCTCGTCGGGATCA
Os09g0441900	GCACAGATCTTGCCGTCTTT	GGTTTACAGCATGAGCAGCA
Os09g0442700	GGTTTTCTTGCCCATCTCAA	TTTGGGCATGAATTTTCTCC
Os09g0442900	CCCTTGCAACAAAAGAGGAA	CCCAAGACATCCTTCCTTCA
OsPIN1	GAAGGACAGGGAGGACTACG	TGAGGCTGGAGTAGGTGTTC
LAZY1	GCCACTGGATCAAGACTGA	TGTAACAGCAAGCACATATTC
qHOX1	AGCACAACACCCTCAATC	GTTCTGGAACCACACCTC
qHOX28	CATTGACCACCCTCACAA	GAATCCGCACAAGAACTG
TAC1	GAGATGGCTCTAAAGGTGTTC	CGTGCCAATTGCAAGTATACC
TAC4	AAGGTCGCAAACAAGCAG	AACTGCCAGGAGCAGAGAG
OsActin	CGTCCTCCTGCTTGTTTCTC	TAGGCCGGTTGAAAACTTTG
